# Blutzuckerselbstkontrolle Update 2023

**DOI:** 10.1007/s00508-023-02172-w

**Published:** 2023-04-20

**Authors:** Thomas C. Wascher, Lars Stechemesser, Jürgen Harreiter, Ausschuss Leitlinien

**Affiliations:** 1 1. Medizinische Abteilung, Hanuschkrankenhaus, Wien, Österreich; 2 Universitätsklinik für Innere Medizin I, Paracelsus Medizinische Privatuniversität - Landeskrankenhaus, Salzburg, Österreich; 3 Abteilung für Endokrinologie und Stoffwechsel, Universitätsklinik für Innere Medizin III, Medizinische Universität Wien, Spitalgasse 23, 1090 Wien, Österreich

**Keywords:** Diabetes mellitus, Blutzucker, Selbstkontrolle, Diabetes mellitus, Blood glucose, Self monitoring

## Abstract

Blutzuckerselbstkontrolle trägt zum integrierten Management des Diabetes mellitus bei. Sie sollte für alle Patienten mit Diabetes mellitus zur Verfügung stehen. Die Blutzuckerselbstkontrolle erhöht die Patientensicherheit, die Lebensqualität und verbessert die Blutzuckerkontrolle. Der vorliegende Artikel stellt die Behandlungsvorschläge der österreichischen Diabetesgesellschaft zum Einsatz der Blutzuckerselbstkontrolle dar.

## Abstract (en)

Self monitoring of blood glucose contributes to the integrated management of diabetes mellitus. It, thus, should be available for all patients with diabetes mellitus. Self monitoring of blood glucose improves patients safety, quality of life and glucose control. The current article represents the recommendations of the Austrian Diabetes Association for the use of blood glucose self monitoring according to current scientific evidence.

## Grundsatz Statement

Blutzuckerselbstkontrolle (BZSK) soll für jeden Patienten mit Diabetes mellitus zur Verfügung stehen. Durch BZSK werden folgende Ziele verfolgt:

I) Reduzierte Spätkomplikationen (Senkung des HbA_1c_)
II) Erhöhte Sicherheit (Reduktion von Hypoglykämien)
III) Verbesserte Krankheitsbewältigung (Empowerment)

Basis einer adäquaten BZSK ist eine entsprechende Schulung des Patienten. Die Befähigung zur Selbstkontrolle sollte auf jährlicher Basis überprüft werden. Die BZSK sollte integrierter Bestandteil im Rahmen der Schulungs- und Behandlungsinteraktionen zwischen Patienten und Diabetesteam sein.

## Strukturierte Blutzuckertagesprofile und laufende Therapiekontrolle

Jede Form von Blutzuckertagesprofil und laufender Therapiekontrolle sollte strukturiert sein. Es sollten dabei sowohl prä- wie postprandiale Werte erfasst werden. Strukturierte Blutzuckertagesprofile dienen dabei primär der Information über den individuellen Tagesverlauf der Glykämie. Diese Dynamik des Blutzuckers kann durch eine regelmäßige Kontrolle des HbA_1c_-Wertes nicht erfasst werden. Weiters erhält der Patient über die BZSK unmittelbare Rückmeldungen über den Einfluss von Ernährung und Bewegung auf seine Blutzuckereinstellung. Dies kann das Verständnis für die Erkrankung und die empfohlene Therapie verbessern. Die Messzeitpunkte und Häufigkeit der Messung für die laufende Therapiekontrolle hängen von der Art der antihyperglykämischen Therapie, deren Hypoglykämiepotential und der Notwendigkeit von Selbstanpassungen ab. Bei Neudiagnose und in Phasen der Einstellung oder Umstellung der Therapie kann eine engmaschigere BZSK sinnvoll sein. In Tab. 1 sind strukturierte Blutzuckertagesprofile unterschiedlicher Intensität dargestellt. Durch vorgegebene Messzeitpunkte, Integration in Behandlungsalgorithmen und Kombination mit Ernährungs- und Bewegungsprotokollen werden für Patienten und behandelndes Diabetesteam sowohl erhöhter Informationsgewinn als auch verbesserte Krankheitsbewältigung ermöglicht.

**Table 1:** 

	Früh vor	Nach	Mittags vor	Nach	Abends vor	Nach	Nachts
*Niedrige Intensität*							
Montag	X	X	–	–	–	–	–
Di	–	–	–	–	–	–	–
Mi	–	–	X	X	–	–	–
Do	–	–	–	–	–	–	–
Fr	–	–	–	–	X	X	–
Sa	–	–	–	–	–	–	–
So	X	X	–	–	–	–	–
*Mittlere Intensität*							
Montag	X	X	–	–	–	–	–
Di	–	–	X	X	–	–	–
Mi	–	–	–	–	X	X	–
Do	X	X	–	–	–	–	–
Fr	–	–	X	X	–	–	–
Sa	–	–	–	–	X	X	–
So	X	X	–	–	–	–	–
*Hohe Intensität*							
Montag	X	X	X	X	X	X	(X)
Di	X	X	X	X	X	X	(X)
Mi	X	X	X	X	X	X	(X)
Do	X	X	X	X	X	X	(X)
Fr	X	X	X	X	X	X	(X)
Sa	X	X	X	X	X	X	(X)
So	X	X	X	X	X	X	(X)

Strukturierte Tagesprofile sollen bei Nichterreichen der Zielwerte entweder den Patienten unmittelbar zu Therapieanpassungen befähigen oder später bei der Kontrolle gemeinsam mit dem Diabetesteam zu Therapieänderungen führen. Für die Dokumentation der Blutzuckertagesprofile stehen herkömmliche Tagebücher in Papierform oder elektronische Lösungen zur Verfügung. Eine statistische und graphische Aufarbeitung der Blutzuckerprofile mittels spezifischer PC-Programme, Smartphone-Applikationen und innovativer Blutzuckermessgeräte kann das Erkennen pathologischer BZ-Muster erleichtern, bei der Entscheidung über Therapieanpassungen helfen und die Kommunikation zwischen Patient und Diabetesteam unterstützen.

## BZSK bei Hypoglykämieverdacht und in Ausnahmesituationen

Grundsätzlich sollte jeder Verdacht einer Hypoglykämie, d. h. jede Hypoglykämiesymptomatik durch eine Blutzuckermessung überprüft werden. Nur so lassen sich Pseudohypoglykämien ausschließen. Akut-intermittierende Erkrankungen, physische und psychische Ausnahmesituationen sollten in ihrer Auswirkung auf den Blutzucker durch BZSK überprüft werden. Die Resultate der BZSK setzen Patienten in die Lage rechtzeitig Therapien anzupassen bzw. das betreuende Diabetesteam zu kontaktieren.

## Harnzuckerselbstkontrolle als möglicher Ersatz zur BZSK?

Die Harnzuckerkontrolle kann keinesfalls als Alternative zur BZSK gesehen werden und hat keinen Stellenwert in der Selbstkontrolle. Sie reflektiert nicht den aktuellen Blutzucker sondern ein Summenphänomen seit der letzten Entleerung der Harnblase, beeinflusst durch die individuelle Nierenschwelle. In der Schwangerschaft ist die Harnzuckerkontrolle durch eine Änderung der Nierenschwelle nicht verwertbar. Zusätzlich wird die Harnzuckermenge auch medikamentös beeinflusst. Bei der Einnahme von SGLT-2-Hemmern wird eine Glukosurie therapeutisch induziert, wodurch die Harnzuckerkontrolle gänzlich ihre Aussagekraft verliert. Im Hinblick auf die o. a. differenzierten Aufgaben der BZSK ist evident, dass diese keinesfalls durch eine Harnzuckerkontrolle erfüllt werden können.

## Blutzuckerselbstkontrolle versus Blutzuckertagesprofil beim Arzt

Blutzuckertagesprofile beim Arzt haben jedenfalls Funktionen die über die reine Messung des Blutzuckers hinausgehen und den Bereich der Arzt-Patienten-Interaktion berühren. Andererseits können sie die eigentlichen Aufgaben der BZSK nur unvollständig erfüllen. Damit sollte die regelmäßige Messung von Blutzuckertagesprofilen beim Arzt für jene Patienten vorbehalten bleiben die nicht in der Lage sind eine adäquate BZSK durchzuführen.

## Kontinuierliche Glukosemessung (s. a. Leitlinie Diabetestechnologie)

Zur kontinuierlichen Glukosemessung stehen grunsätzlich Systeme zur Verfügung die in Echtzeit arbeiten (rtCGM) und solche die intermittierend ausgelesen werden müssen (isCGM).

### rtCGM

Technisch steht die Möglichkeit des kontinuierlichen Glukosemonitorings in Echtzeit in der interstitiellen Gewebsflüssigkeit über mehrere Tage von Seiten unterschiedlicher Anbieter zur Verfügung. Die Daten der Messung werden in Echtzeit an ein Endgerät (Systemempfänger, Mobiltelefon, Smartwatch) des Trägers übertragen.

### isCGM

Anders als beim rtCGM handelt es sich bei isCGM (aktuell Abbott Freestyle Libre) nicht um ein Echtzeit-System – vielmehr müssen die Daten zumindest alle 8 h ausgelesen werden um ein komplettes 24 Stundenprofil des Glukoseverlaufes zu erhalten.

Indikationen für den kurzfristigen, diagnostischen Einsatz von CGM sind etwa der Verdacht auf nächtliche, nicht wahrgenommene Hypoglykämien, oder unerklärlich stark schwankende Blutzuckerwerte. Indikationsstellung, Durchführung und kontinuierliche Betreuung sollten an Schwerpunkteinrichtungen erfolgen.

Im Zusammenhang mit einer intensivierten Insulintherapie oder einer Therapie mittels CSII (Pumpentherapie) stellt das CGM im dauerhaften Einsatz eine wichtige, die Patientensicherheit und den Therapieerfolg fördernde technische Möglichkeit dar. Im Vergleich zur blutigen Punktmessung werden wesentlich umfangreichere und strukturiertere Informationen generiert, die einer strukturierten Auswertung und Analyse unterzogen werden sollten.

## Struktur und Häufigkeit der BZSK

Die Struktur und Häufigkeit der Messungen hängt dabei in erster Linie von der Art der antihyperglykämischen Therapie ab. Tab. 2 fasst die unterschiedlichen Indikationen im Rahmen der BZSK zusammen und gibt eine Näherung an die dazu durchschnittlich notwendigen Messungen pro Monat an. Für Patienten welche eine intensivierte Insulintherapie oder Insulinpumpentherapie haben kann auf Grund individueller Situationen (Sport, häufige Hypoglykämien, Gastroparese) eine höhere Messintensität notwendig sein. Eine Sonderstellung nimmt der Gestationsdiabetes ein. Betroffene Patientinnen sollen den Blutzucker zumindest nüchtern und 1 h nach den Hauptmahlzeiten – also mindestens 4 × pro Tag – unabhängig von der Therapiemodalität überprüfen.

**Table 2:** 

Kontrollsituation und Blutzuckerkontrollen pro Monat, ca.	A (5)	H (10–15)	STP (25–30)	TK (30–150)	Kontrollen/Monat
Ausschließlich Diät	X	–	X	–	30
OADs ohne Hypoglykämiepotenial	X	–	X	–	30
OADs mit Hypoglykämiepotential	X	X	X	(X)	45 (–75)
Insulin (± OAD) ohne Selbstanpassung	X	X	X	X	75
Insulin (± OAD) mit Selbstanpassung	X	X	X	XX	105
Intensivierte Insulintherapie oder Pumpe	X	XX	X	XXX	200 (–250)

*A* Kontrolle bei Ausnahmesituationen (interkurrente Krankheit etc.), *H* Hypoglykämieüberprüfung, *STP* Strukturierte Blutzuckertagesprofile, *TK* kontinuierliche Therapiekontrolle

## Evidenzlage

Patienten mit einer intensivierten Insulintherapie oder einer Insulinpumpentherapie profitieren in zahlreichen Studien von BZSK. Die strukturierte und ausreichend häufige BZSK kann – als Bestandteil einer entsprechenden Schulung – die glykämische Kontrolle und die glykämische Variabilität bei diesen Patienten verbessern [1, 2].

Die Studienlage über den Nutzen von BZSK, insbesondere bei Patienten mit Typ‑2 Diabetes ohne Insulintherapie, ist weniger eindeutig. Bei Patienten mit Typ‑2 Diabetes die mit Insulin behandelt werden scheint, so wie bei Patienten mit Typ‑1 Diabetes, eine höhere Intensität der BZSK, gemessen an der Zahl der täglichen Kontrollen, mit einem niedrigeren HbA_1c_ assoziiert zu sein [3, 4]. In der selben Querschnittsuntersuchung konnte dies für Patienten mit Typ‑2 Diabetes unter oralen Antidiabetika nicht gezeigt werden. Ein Cochrane Review aus dem Jahr 2005 [5] kommt zu dem Schluss, dass die Datenlage zwar einen bescheidenen Effekt der BZSK auf das HbA_1c_ bei Patienten mit Typ‑2 Diabetes ohne Insulintherapie vermuten lässt, weitere Studien allerdings dringend notwendig wären (Evidenzklasse A). In einer weiteren Metaanalyse findet Jansen [6], dass bei Patienten unter oraler Therapie nur eine sogenannte „Feedback kontrollierte“ BZSK in einem gering aber signifikant verbesserten HbA1c resultiert (Evidenzklasse A). Dies bedeutet, dass erst eine adäquate Reaktion auf die BZSK potenziell die glykämische Kontrolle verbessern kann. Dabei wird das Resultat in erster Linie von einer Studie [7] getrieben. Ein dazu divergierendes Studienresultat wurde aus England berichtet. Farmer et al. [8] konnten trotz Feedback Kontrolle keinen Zusammenhang zwischen BZSK und HbA1c feststellen. Andererseits zeigen auch die retrospektive ROSSO Studie [9] wie auch eine große (*n* = 610), randomisierte Studie [10] günstige Effekte der BZSK auf das HbA1c. Sowohl ein Cochrane Review als auch eine Metaanalyse (beide 2012 veröffentlicht) kommen zu dem Schluss, dass bei Patienten mit Typ‑2 Diabetes ohne Insulintherapie der Effekt der BZSK auf das HbA1c nach einer Diabetesdauer von mehr als 1 Jahr moderat ist ([11, 12], Evidenzklasse A). Einschränkend dazu muss jedoch angemerkt werden, dass Patienten bereits bei Diagnosestellung hinsichtlich BZSK zu schulen sind und die zuletzt genannte Analyse daher nur eingeschränkt alltagstaugliche Bedeutung hat.
